# Dry Eye Disease Prevalence and Associated Risk Factors Among the Middle East Population: A Systematic Review and Meta-Analysis

**DOI:** 10.7759/cureus.70522

**Published:** 2024-09-30

**Authors:** Zoelfigar Mohamed, Saif Alrasheed, Mustafa Abdu, Kareem Allinjawi

**Affiliations:** 1 Department of Optometry, College of Health Sciences, University of Buraimi, Buraimi, OMN; 2 Department of Optometry, College of Applied Medical Sciences, Qassim University, Buraydah, SAU; 3 Department of Optometry, College of Applied Medical Sciences, University of Jeddah, Jeddah, SAU

**Keywords:** dry eye, global health, middle east, prevalence, risk factors

## Abstract

Dry eye disease (DED) is a common condition characterized by a loss of the tear film function, leading to symptoms of ocular discomfort and damage to the ocular surface. The prevalence and associated risk factors of DED may vary by region due to environmental, cultural, and genetic differences. The aim of the present study is to systematically review and analyze the prevalence and risk factors of DED in the Middle East (ME) region.

This study adhered to the PRISMA 2020 guidelines. A comprehensive literature search was conducted using databases such as Web of Science, Scopus, Google Scholar, and PubMed to identify relevant studies published from January 2004 to July 2024. Studies included in the review were those that provided data on the prevalence and risk factors of DED in Middle Eastern populations. Data were extracted and analyzed to determine overall pooled prevalence and associated risk factors using a random-effects model. The study protocol was registered in the International Prospective Register of Systematic Reviews (PROSPERO) with the registration number CRD42024583897.

The meta-analysis included 17 studies from 10 countries with a total of 22,087 subjects. The estimated pooled prevalence of DED in the ME region was 28.33% (95% CI: 27.74-28.93). The primary risk factors include age, female gender, and smoking. Other risks are contact lens use, prolonged screen time (over six hours daily), diabetes, glaucoma medications, allergies, autoimmune diseases, refractive surgery, arthritis, high cholesterol, acne treatments, antihistamines, antidepressants, thyroid disease, and a history of conjunctival infections or corneal abrasions. The prevalence of DED in Middle Eastern countries was higher than the global estimate, highlighting significant regional variation. Common risk factors for DED include older age, female gender, and smoking. These findings underscore the need for targeted prevention and management strategies that address the specific risk factors prevalent in the Middle Eastern population.

## Introduction and background

Dry eye disease (DED) is a condition that arises from an imbalance in the tear film, either from a lack of tears or excessive evaporation. The condition causes discomfort and damage to the surface of the eye, particularly in the area between the eyelids [1]. DED is the most widespread ocular surface disorder, primarily caused by inadequate or unstable tear production. Some types of DEDs are characterized by aqueous tear deficiency, as seen in Sjögren's syndrome. However, previous studies [2-4] indicate that numerous factors can contribute to tear film instability. Since DED is considered a multifactorial condition, it leads to a range of ocular complaints and visual disturbances, which can ultimately result in damage to the surface of the eye [5]. Currently, there is no consensus on the diagnostic criteria or classification of disease states for DED, despite the various pathogenic factors involved [6]. Additionally, individuals with DED often experience a reduced quality of life, particularly when performing near-vision tasks or working on video terminals, which can impose restrictions on their daily activities and living environment [7].

DED is recognized as a growing global public health problem [8]. A recent systematic review and meta-analysis estimated the global prevalence of DED to be approximately 11.59% [9]. However, DED is reported to be notably higher among people in Africa, with a prevalence of 42.0% [7], and 20.1% in Asia [10]. The increasing incidence of DED merits greater attention from eye care professionals, as it is now affecting nearly one in five adults [11,12]. Estimates of the condition may vary based on factors such as ethnicity, residential environment, lifestyle habits, and the diagnostic criteria used [13-15]. Previous studies [16-18] have identified the most common risk factors for DED as advanced age, contact lens wear, refractive surgery, medication use, and systemic diseases.

The Middle East (ME) region comprises 17 countries, spanning from Iran in the east to Egypt in the west, Yemen in the south, and Turkey in the north [19]. The prevalence of DED varies significantly across these countries, with rates reported as high as 62.6% in Palestine [20] and as low as 8.3% in Turkey [21]. Many studies in this context have been limited by small sample sizes and varying criteria. Additionally, there is a lack of comprehensive data on the overall prevalence and risk factors of DED in the ME region. Therefore, this systematic review and meta-analysis aims to estimate the prevalence and identify the risk factors of DED in ME populations. Such information is essential for developing effective public health strategies. The study provides a thorough overview of DED prevalence and highlights key risk factors in the ME region.

## Review

Methods

Search Strategy and Study Design

The present study followed the Preferred Reporting Items for Systematic Reviews and Meta-Analyses (PRISMA 2020) framework [22]. The review protocol was registered in the International Prospective Register of Systematic Reviews (PROSPERO) with the registration number CRD42024583897. The authors searched Web of Science, Scopus, and Google Scholar for studies on the prevalence and risk factors of DED in the ME region published between January 2004 and July 2024. The authors conducted a search using Medical Subject Headings (MeSH) terms, including: (rate, prevalence, frequency, incidence, distribution, proportion, OR epidemiology), (risk factors OR contributing factors OR determinants OR causes OR influencing factors) AND dry eye disease. Additionally, searches were refined using AND/OR operators to focus on studies from Middle Eastern countries, including Bahrain, Egypt, Iran, Iraq, Jordan, Kuwait, Lebanon, Oman, Palestine, Qatar, Saudi Arabia, Syria, Sudan, Turkey, Tunisia, the United Arab Emirates, and Yemen.

Selection Criteria

**Figure 1:**
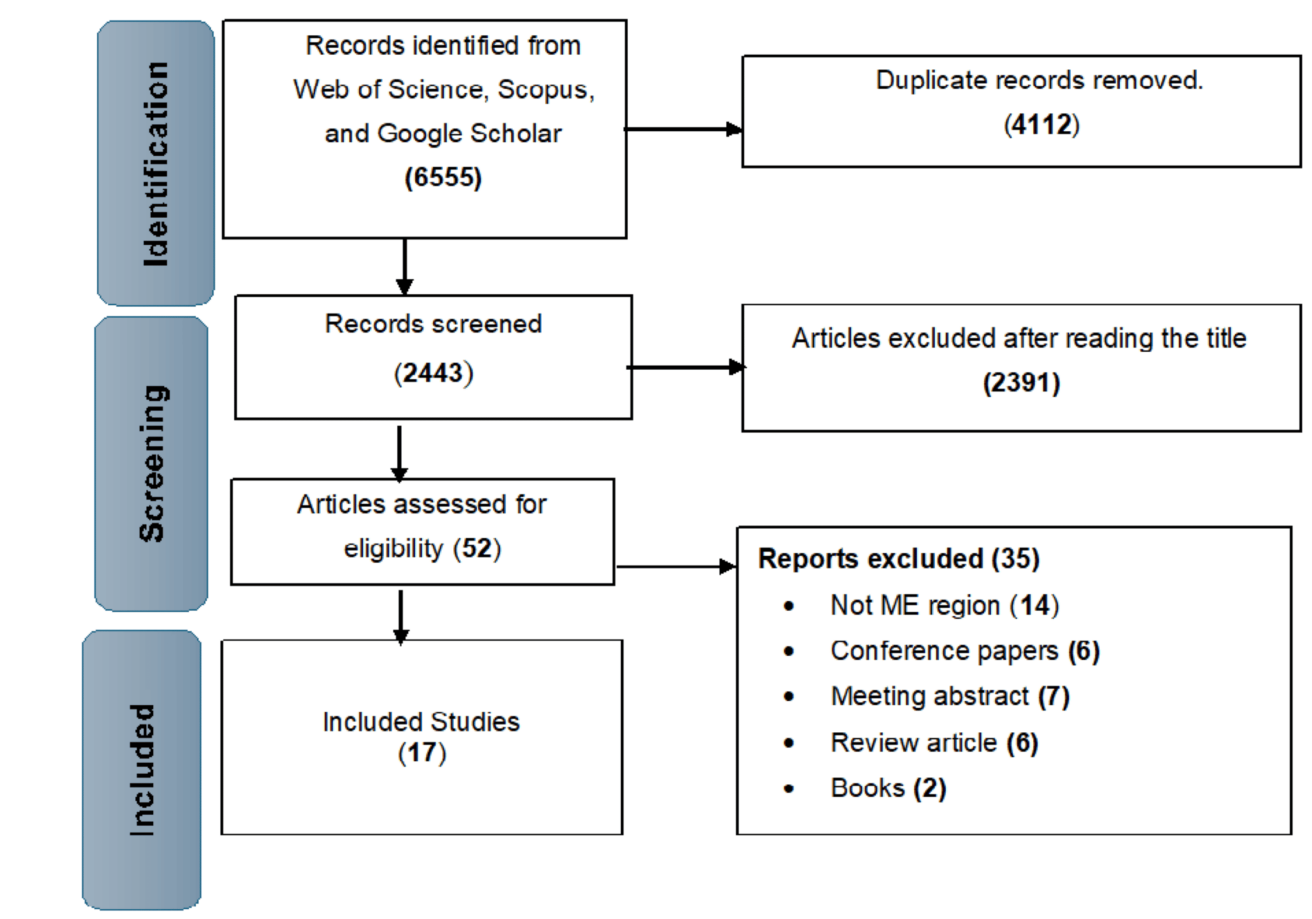
Identification of studies included in this review

This systematic review and meta-analysis focused on studies published in English, available online, in peer-reviewed journals, and assessing the prevalence and risk factors of DED in the ME region. Inclusion criteria required studies to involve at least 100 individuals from a Middle Eastern population and to be population-based, cohort, cross-sectional, or randomized controlled trials. Studies were excluded if they were not conducted in the ME or did not address the prevalence and/or risk factors of DED. Additionally, conference papers, editorial discussions, meeting abstracts, and studies lacking basic data collection were excluded from this review, as shown in Figure 1. The quality of each selected study was individually assessed using a checklist created by Downs and Black, with each article receiving a score based on a 10-item evaluation; this reflects the average checklist score after the individual assessment [23], as shown in Table 1 [20-39].

**Table 1:** Quality assessment of included studies in systemic review and met analysis, according to the checklist created by Downs and Black The quality of each selected study included in this systematic review and meta-analysis was assessed using a checklist created by Downs and Black, with each article receiving a score based on a 10-item evaluation [23]. 0 indicates studies that did not report a predefined checklist; 1 indicates studies that reported a predefined checklist

First author-year	10-item for evaluation										Quality score
	Objective	Outcomes	Characteristics	Sample size	Findings	p-value	Data cleaning	Variability	Statistical tests	Validity	
Shanti et al. 2020 [20]	1	1	1	1	1	1	1	1	1	1	10
Yilmaz et al. 2015 [21]	1	1	1	1	1	1	1	0	1	1	9.0
Alshamrani et al. 2017 [25]	1	1	1	1	1	1	1	1	1	1	10
Yasir et al. 2019 [26]	1	1	1	1	1	1	1	0	1	1	9.0
Helayel et al. 2023 [27]	1	1	1	1	1	1	1	1	1	1	10.0
Alhamyani et al. 2017 [28]	1	1	1	0	1	1	1	1	1	1	9.5
Alkhaldi et al. 2022 [29]	1	1	1	0	1	1	1	1	1	1	9.0
Alkabbani et al. 2021 [30]	1	1	1	0	1	1	1	1	1	1	9.0
Dossari et al. 2022 [31]	1	1	1	1	1	1	1	1	1	1	10
Mourad et al. 2018 [32]	1	1	1	0	1	0	1	1	1	1	8
Rashwan et al. 2019 [33]	1	1	0	0	1	1	1	1	1	1	8
Iqbal et al. 2018 [34]	1	1	0	1	1	1	1	1	1	1	9.0
Haddad et al. 2017 [35]	1	1	1	0	1	1	1	1	1	1	9.0
Younis et al. 2019 [36]	1	1	1	0	1	0	1	1	1	1	8
Zbiba et al. 2018 [37]	1	1	1	0	1	1	1	1	1	1	9.0
Sherry et al. 2019 [38]	1	1	1	1	1	1	1	1	1	1	10
Omer et al. 2013 [39]	1	1	1	0	1	1	1	1	1	1	9

Data Extraction

After evaluating the titles and abstracts, the authors ZM and SA reviewed the full content of each selected study. They used a standardized form to record data, including the first author, year of publication, country of the study, sample size, age, diagnostic criteria used in selected studies, prevalence, and risk factors for developing DED. The approved diagnostic criteria used in the present study include the presence of at least one ocular manifestation, determined through evaluation of tear film stability, osmolality, or ocular surface damage, in conjunction with a positive result gathered from validated assessment tools, such as the 5-item Dry Eye Questionnaire (DEQ-5) or the Ocular Surface Disease Index (OSDI) [24]. In the present systematic review and meta-analysis, clear standards and protocols were developed to address disagreements between authors and guide the review process. These protocols facilitated open communication, enabling authors to present different viewpoints and reach a consensus by referring to the established guidelines.

Data Analysis

The meta-analysis was conducted using MedCalc version 19.6.1 (MedCalc Software, Ostend, Belgium). Data were entered individually from a predesigned format that included the author's name, publication date, study population, mean age, study design, sample size, diagnostic methods, as well as the prevalence and risk factors of DED. Heterogeneity among the selected studies was evaluated using a Q statistic, distributed as χ² under the assumption of homogeneous effect sizes, and the I² index (0-75%), indicating the level of heterogeneity from none to high. The analyzed data showed the prevalence of DEDs with the corresponding weight for each study. The overall pooled prevalence of DED was estimated using a random-effects model and its associated 95% confidence intervals (CIs). Furthermore, Begg's and Egger's tests were employed to estimate the publication bias of the articles encompassed. A p-value of less than 0.05 was judged statistically significant for all analyses.

Results

Characteristics of Articles Reporting the Proportion of DED

The initial search yielded 6,555 articles (Figure 1). These studies were screened for eligibility by excluding non-English publications and removing duplicates. The abstracts and titles of the remaining studies were then inspected, and any articles that did not meet the review inclusion criteria were excluded.

The meta-analysis investigating the prevalence of DED included 17 studies from 10 countries, as shown in Table 2 [20-39]. These studies were published between 2013 and 2023 and comprised 22,087 subjects aged 17 years or older.

**Table 2:** Characteristics of the articles included in the systematic review and meta-analysis DED: dry eye disease

First author-year	Country	Study design	Diagnostic methods	Sample	Positive cases of DED	Age (mean)
Alshamrani et al. 2017 [25]	Saudi Arabia	Cross sectional	Symptoms survey	1858	596	39.3
Yasir et al. 2019 [26]	Saudi Arabia	Cross sectional	Symptoms survey	890	401	≥40
Helayel et al. 2023 [27]	Saudi Arabia	Cross sectional	Symptoms survey	2042	784	≥18
Alhamyani et al. 2017 [28]	Saudi Arabia	Cross sectional	Symptoms survey	482	366	50.16
Alkhaldi et al. 2022 [29]	Saudi Arabia	Cross sectional	Symptoms survey	4066	2016	38.7
Alkabbani et al. 2021 [30]	UAE	Cross sectional	Symptoms survey	452	283	17-40
Dossari et al. 2022 [31]	Saudi Arabia	Cross sectional	Symptoms survey	1381	242	32.53
Mourad et al. 2018 [32]	Egypt	Hospital based- Prospective	Clinical diagnosis	6252	625	46.51
Rashwan et al. 2019 [33]	Egypt	Hospital based - prospective	Clinical diagnosis	500	64	NA
Iqbal et al. 2018 [34]	Egypt	Cross sectional	Symptoms survey	100	28	≥18
Shanti et al. 2020 [20]	Palestine	Cross sectional	Both	769	492	43.61
Haddad et al. 2017 [35]	Jordan	Cross sectional	Symptoms survey	802	305	28
Younis et al. 2019 [36]	Iraq	Cross sectional	Clinical diagnosis	103	28	41.5
Zbiba et al. 2018 [37]	Tunisia	Hospital based- retrospective	Clinical diagnosis	230	66	≥20
Sherry et al. 2019 [38]	Lebanon	Cross sectional	Symptoms survey	602	219	≥18
Omer et al. 2013 [39]	Sudan	Hospital based- prospective	Clinical diagnosis	100	32	43.6
Yilmaz et al. 2015 [21]	Turkey	Cross sectional	Symptoms survey	1458	121	41

Prevalence of DED in the ME Region

A total of 17 studies reported the prevalence of DED in the ME population, with occurrence ranging from 8.29% to 62.61%. These studies were conducted in 10 countries and included patients from Saudi Arabia, Egypt, Tunisia, Iraq, the UAE, Sudan, Lebanon, Jordan, Palestine, and Turkey.

**Figure 2:**
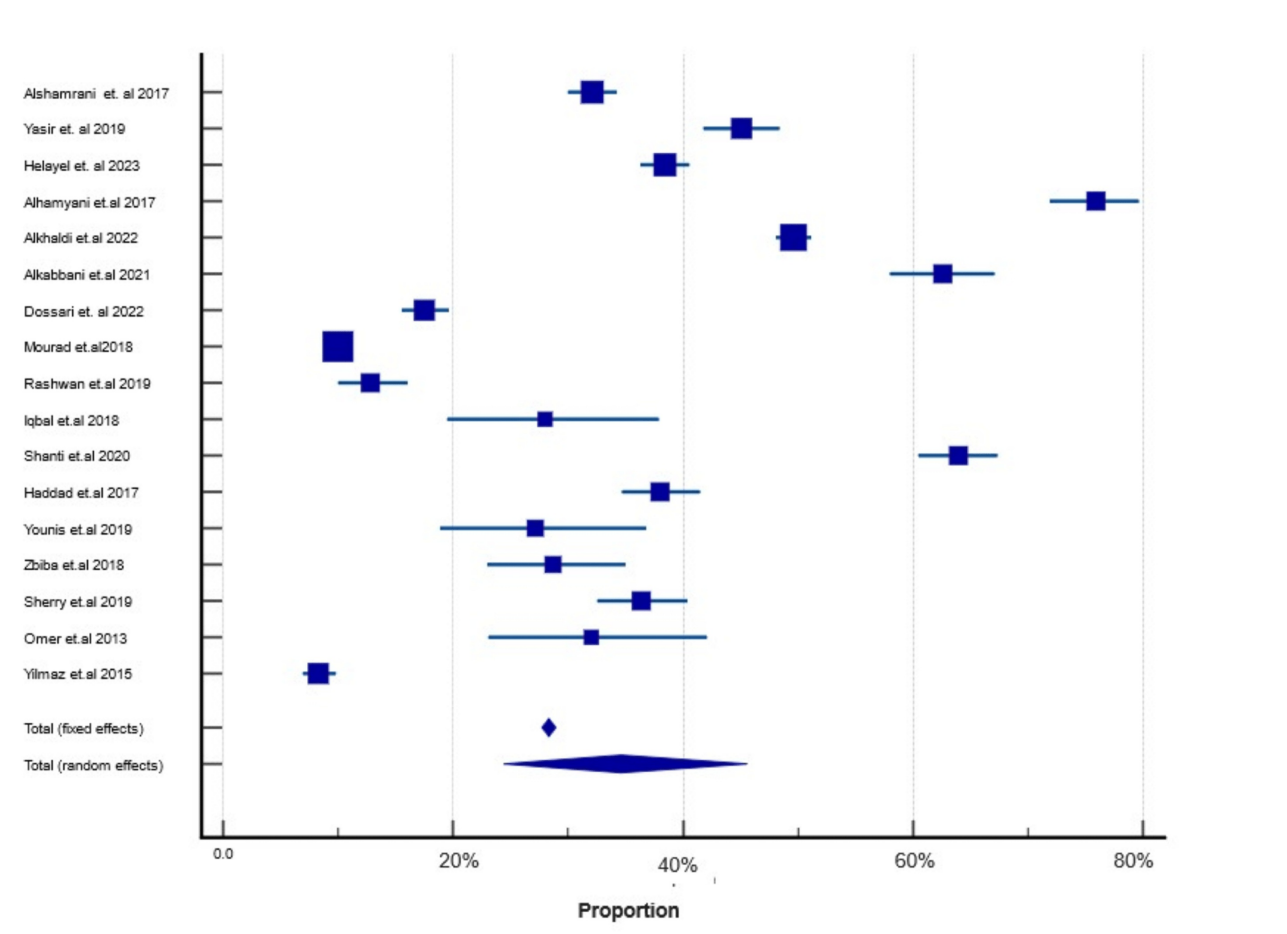
Forest plots showing pooled prevalence of DED in the ME region DED: dry eye disease; ME: Middle East

The pooled prevalence of DED in the ME region was 28.33% (95% CI: 27.74-28.93; p < 0.001). According to the current review of 11 studies, approximately 64.7% reported a significantly higher prevalence of DED, while 35.3% (n = 6) reported a lower prevalence compared to the pooled estimated prevalence. The study conducted by Shanti et al. found that the Palestinian population had the highest prevalence of DED at 63.98% (95% CI: 60.47 to 67.38), with a mean age of 43.61 years [20]. In contrast, Yilmaz et al. reported the lowest prevalence among the Turkish population, at 8.30% (95% CI: 6.93 to 9.86), with a mean age of 41 years [21]. The degree of heterogeneity between the studies included in this meta-analysis was highly significant (p < 0.0001), as shown in Table 3 and Figure 2.

**Table 3:** The prevalence of DED in ME region with confidence interval (CI) Weight (%) indicates the percentage of study weights in a meta-analysis, showing the contribution of each study to the overall summary results; heterogeneity between studies is indicated by p < 0.001 I^2^ (inconsistency) = 99.62% DED: dry eye disease; ME: Middle East

Study	Sample size	Prevalence (%), 95% CI	Weight (%)
Alshamrani et al. 2017 [25]	1858	32.08 (29.96 to 34.25)	8.41
Yasir et al. 2019 [26]	890	45.06 (41.75 to 48.39)	4.03
Helayel et al. 2023 [27]	2042	38.39 (36.28 to 40.54)	9.24
Alhamyani et al. 2017 [28]	482	75.93 (71.86 to 79.69)	2.19
Alkhaldi et al. 2022 [29]	4066	49.58 (48.03 to 51.13)	18.40
Alkabbani et al. 2021 [30]	452	62.61 (58.00 to 67.09)	2.05
Dossari et al. 2022 [31]	1381	17.52 (15.55 to 19.63)	6.25
Mourad et al. 2018 [32]	6252	10.00 (09.26 to 10.77)	28.29
Rashwan et al. 2019 [33]	500	12.80 (10.00 to 16.05)	2.27
Iqbal et al. 2018 [34]	100	28.00 (19.48 to 37.87)	0.46
Shanti et al. 2020 [20]	769	63.98 (60.47 to 67.38)	3.48
Haddad et al. 2017 [35]	802	38.03 (34.66 to 41.49)	3.63
Younis et al. 2019 [36]	103	27.18 (18.88 to 36.84)	0.47
Zbiba et al. 2018 [37]	230	28.70 (22.94 to 35.01)	1.05
Sherry et al. 2019 [38]	602	36.38 (32.53 to 40.36)	2.73
Omer et al. 2013 [39]	100	32.00 (23.02 to 42.08)	0.46
Yilmaz et al. 2015 [21]	1458	08.30 (06.93 to 09.86)	6.60
Total	22087	28.33 (27.74 to 28.93)	100.00

Publication Bias

Egger's and Begg's tests were conducted to investigate publication bias. The results of Begg's test (p = 0.967) and Egger's test (p = 0.215) suggested no significant bias. Therefore, no substantial evidence of publication bias related to the occurrence of DED in the ME region was observed. However, it was not possible to examine the impact of publication bias on the prevalence and risk factors of DED due to the limited number of available studies.

Risk Factors of DED in the ME Region

Nine studies discussed the risk factors of DED in the ME region, as shown in Table 4, six of which reported that increasing age increased the likelihood of DED occurrence [20,24,27,29,30,31,38]. Seven studies investigated the association between the prevalence of DED and sex, revealing that the disease is more prevalent in females [2,24-27,29-31]. Four studies reported that smoking increased the incidence of dry eyes [24,26,30,38]. In addition, other factors reported included contact lens wearers, high daily screen time (>6 hours), diabetes, glaucoma eye drops, allergy, autoimmune disease, refractive surgery, arthritis, hypercholesterolemia, acne treatment, antiallergy drugs, antidepressants, thyroid disease, rheumatoid arthritis, use of antihistamines, history of conjunctival infection, and history of corneal abrasions [24-31,38].

**Table 4:** Risk factors of dry eye disease in the Middle East region

First author-year	Country	Sample	Risk factors
Alshamrani et al. 2017 [25]	Saudi Arabia	1858	Females, age, smoking, and diabetics
Yasir et al. 2019 [26]	Saudi Arabia	890	Females, glaucoma eye drops
Helayel et al. 2023 [27]	Saudi Arabia	2023	Age, females, diabetes, allergy, autoimmune disease, smoking, and history of refractive surgery
Alhamyai et al. 2017 [28]	Saudi Arabia	482	Smoking, diabetes, arthritis, and hypercholesterolemia
Alkhaldi et al. 2022 [29]	Saudi Arabia	4066	Females, age, refractive surgery, acne treatment, contact lens wearers, antiallergy drugs, antidepressants, and computer use of >6 hours per day.
Alkabbani et al. 2021 [30]	UAE	452	Females, age, smoking, excessive daily screen time, and contact lenses.
Dossari et al. 2022 [31]	Saudi Arabia	1381	Female gender, electronic devices for a continued time, contact lens, eye surgery, conjunctival infection, corneal abrasions systemic lupus, thyroid disease, rheumatoid arthritis, antidepressants, antihistamine
Shanti et al. 2020 [20]	Palestine	769	Female and older age
Sherry et al. 2019 [38]	Lebanon	602	Smokers, age

Discussion

This systematic review and meta-analysis were conducted to estimate the prevalence and identify risk factors for developing DED among the ME population. To the best of our knowledge, this systematic review and meta-analysis is the first to provide an overall estimate of DED prevalence in the Middle East. Environmental, sociodemographic, and medical factors significantly influence the development of DED, which can impact well-being and quality of life [39]. The second part of this review systematically analyzed the relationship between various risk factors and the development of DED, with the goal of creating a predictive model to prevent and detect DED early, thereby minimizing its complications.

Seventeen studies met our criteria for investigating the prevalence and risk factors for developing DED in the ME region. The findings revealed that the overall prevalence of DED in the ME was 28.33%, with a confidence interval of 27.74 to 28.93, based on a total population of 22,087. This prevalence corresponds to the global range for DED, which is 5% to 50%, according to a systematic review conducted by Stapleton et al. in 2017 [40]. The prevalence reported in the ME is higher than that in the US adult population (6.8%) [41], the Canadian population (22.0%) [42], and the Asian population (20.1%) [43], but lower than in the African region [8]. The widespread use of air conditioners in the ME due to hot weather may contribute to the high prevalence of DED. Other environmental factors, such as wind, dry climate, and air pollution, may also play a role in increasing the prevalence of DED in this region.

The current study reviewed the risk factors for developing DED in the ME region. Several previous studies reported that DED increases with age [44,45], which was consistent with this systematic review. The current review also showed that women are more prone to DED in the ME region. These findings confirm the results of previous reports showing higher DED prevalence among women than men [44,46,47] and confirm that gender is a powerful factor in the development of DED in the ME region. The prevalence of dry eye symptoms among women highlights the importance of sex hormones, particularly androgens, which affect the lacrimal glands and ocular surface sensitivity [17].

This review showed that smoking increased the incidence of DED. While most research has found that smoking is not a significant risk factor [48], some studies have reported a significant association between smoking and DED [49]. Additionally, other factors have been suggested to correlate with DED in the ME region. Our study found that factors such as contact lens wear, high daily screen time (>6 hours), diabetes, use of glaucoma eye drops, allergies, autoimmune diseases, refractive surgery, arthritis, hypercholesterolemia, acne treatment, use of antiallergy drugs, antidepressants, thyroid disease, rheumatoid arthritis, use of antihistamines, history of conjunctival infection, and history of corneal abrasions increase the risk of developing DED. These factors have been confirmed by previous research conducted among various study populations [17,50,51].

The current review revealed significant variations in DED prevalence among the ME population, ranging from 8.30% to 63.98%. The meta-analysis showed high heterogeneity between studies, indicating p < 0.001. This variation could be attributed to the different diagnostic criteria used in ME studies, which included subjective and objective findings of DED. Such criteria as symptoms-based surveys, tear film abnormalities, and epithelial damage were used in all studies reviewed. Less stringent criteria for symptoms or epithelial damage were associated with a higher prevalence of DED. Therefore, we emphasize the need for standardized diagnostic criteria to accurately estimate DED prevalence and develop preventive measures.

The main limitation of this systematic review was the variation in how dry eye was identified across the included articles. The use of diverse types of measurements and methods may have resulted in heterogeneity of the data. Additionally, many studies were conducted in a limited number of countries, making it uncertain whether the findings can be generalized to all Middle Eastern populations. Furthermore, the severity and progression of dry eye in relation to the risk factors, as well as the differences between symptomatic and diagnostic dry eye, were not considered. Despite these limitations, the current systematic review and meta-analysis provide valuable information about the prevalence and risk factors of DED in the ME region.

## Conclusions

The pooled prevalence of DED in the ME was high compared to the estimated global prevalence of DED, highlighting the need for urgent attention and action as a significant public health concern. There is notable variation in prevalence among different countries in the region and even within the same country, due to differences in geographic characteristics and climate conditions. Associated risk factors for DED include being female, aging, smoking, contact lens wear, high daily screen time (>6 hours), diabetes, use of glaucoma medication, allergies, autoimmune diseases, refractive surgery, arthritis, hypercholesterolemia, acne treatment, use of anti-allergy drugs, antidepressants, thyroid disease, rheumatoid arthritis, use of antihistamines, and history of conjunctival infections or corneal abrasions.
